# Supplementary material for: Dock10 Regulates Cardiac Function under Neurohormonal Stress
Source: Int J Mol Sci. 2022 Aug 25;23(17):9616. doi: 10.3390/ijms23179616 (PMC9455810; doi:10.3390/ijms23179616)
Supplement: Supplementary file 1 [file ijms-23-09616-s001.zip › ijms-1835772-supplementary.pdf]

SUPPLEMENTAL MATERIAL

Liad Segal[1,2], Sharon Etzion[2], Sigal Elyagon[1,2], Moran Shahar[1], Hadar Klapper-Goldstein[1,2], Aviva Levitas[3], Michael S. Kapiloff[6] , Ruti Parvari[4,5] and Yoram Etzion[1, 2]

[1] Department of Physiology and Cell Biology, Ben-Gurion University of the Negev, Beer-Sheva, Israel.

[2] Regenerative Medicine and Stem Cell (RMSC) Research Center, Ben-Gurion University of the Negev, Beer-Sheva, Israel.

[3] Pediatric Cardiology Department, Soroka University Medical Center, Beer-Sheva, Israel.

[4] Department of Microbiology, Immunology and Genetics, Ben-Gurion University of the Negev, Beer-Sheva, Israel.

[5] National Institute for Biotechnology in the Negev, Ben-Gurion University of the Negev, Beer-Sheva, Israel.

[6] Departments of Ophthalmology and Medicine (Cardiovascular Medicine), Stanford Cardiovascular Institute, Stanford University, Palo Alto, CA, USA.

Abbreviated title: Role of Dock10 in the heart

Table S1: Gene primers

mActa1r CCACCGATCCACACTGAGTA	mActa1f AAGTGCGACATCGACATCAG
mActa2r CACCAGGGCTGTGCTGTCTT	mActa2f AGCCAGTCGCTGTCAGGAA
mNppar AATGTGACCAAGCTGCGTGA	mNppaf GCTGCAACAGCTTCCGGTA
MNppbr TGGTCCTTCAAGAGCTGTCTC	MNppbf AGGTGCTGTCCCAGATGATT
mMyh7r TCCACGATGGCGATGTTCT	mMyh7f CCTCCAGAGTCTGCTGAAGGA
mDock10r TCCAGATCCTTGGGGTTTACC	mDock10f CTGACAGGGCTCTCGGAGAT
mDock9r TCCACCCACTTAACCTCTGG	mDock9f AGAAGAAGGACGCTGTGGAA
mDock11r AGCCACATGGAAACTCATCC	mDock11f CGCAGTTGTACCTCCCATTT
mCol1a1r GACGTGCTTCTTTTCCTTGG	mCol1a1f TGACTGGAAGAGCGGAGAGT
mCol3a1r GTCACCATTTCTCCCAGGAA	mCol3a1f CAATATGCCCACAGCCTTCT
mCol1a2r TGGGACCATCAACACCATC	mCol1a2f TGCTCAGCTTTGTGGATACG
mGapdhr CCAATACGGCCAAATCCGT	mGapdhr TCTTGTGCAGTGCCAGCCT

Table S2: Echocardiogrphy of global Dock10 KO mice following chronic exposure to AngII

	WT		KO			
	**Saline** (n=8)	**AngII** (n=8)	**Saline** (n=7)	**AngII** (n=10)	**P value** #	*
Heart rate (b.p.m.)	493.1±14.9	473.0±25.2	493.4±3.1	548.5±17.3	ns	*
LVIDd (mm)	3.94±0.09	4.11±0.15	3.93±0.12	4.13±0.13	ns	ns
LVIDs (mm)	2.54±0.13	2.95±0.18	2.61±0.08	3.25±0.11	ns	**
RWT	0.53±0.02	0.56±0.01	0.68±0.04	0.63±0.06	ns	ns
EF%	64.04±2.55	54.33±3.15	62.60±4.05	44.07±2.25	ns	***
FS%	35.42±2.22	31.40±2.14	33.62±2.85	20.92±0.98	#	***

LVIDd = Left ventricular internal diameter - Diastolic; **LVIDs** = Left ventricular internal diameter- Systolic; **RWT** = Relative wall thickness; **EF** = Ejection fraction; **FS** = Fractional shortening. # Saline vs. AngII in WT mice. *, **, *** Saline vs. AngII in KO mice.

Table S3: Dock10 CKO mice - Gravimetric and echocardiographic data following exposure to AngII

	Cre^{+} (n=10)	Fl/Fl (n=9)	Cre^{+} Fl/Fl (n=11)	P value
HW/BW	5.72±0.15	5.95±0.32	5.90±0.33	ns
HW/TL	6.79±0.31	7.49±0.46	7.36±0.25	ns
Heart rate (b.p.m)	516.22±31.71	504.511±16.93	507.90±22.09	ns
LVIDd (mm)	3.92±0.13	4.08±0.15	4.12±0.10	ns
LVIDs (mm)	3.15±0.16	3.30±0.16	3.32±0.14	ns
RWT	1.02±0.02	1.03±0.05	1.00±0.04	ns
EF%	40.68±3.89	39.27±2.61	39.13±3.34	ns

LVIDd = Left ventricular internal diameter - Diastolic; **LVIDs** = Left ventricular internal diameter- Systolic; **RWT** = Relative wall thickness; **EF** = Ejection fraction. P-value indicate the result of 1 way ANOVA between the 3 groups.
